# Potent Antifungal Activity of Pure Compounds from Traditional Chinese Medicine Extracts against Six Oral *Candida* Species and the Synergy with Fluconazole against Azole-Resistant *Candida albicans*

**DOI:** 10.1155/2012/106583

**Published:** 2012-02-12

**Authors:** Zhimin Yan, Hong Hua, Yanying Xu, Lakshman P. Samaranayake

**Affiliations:** ^1^Department of Oral Medicine and Traditional Chinese Medicine, Peking University School and Hospital of Stomatology, 22 South Zhongguancun Street, Beijing 100081, China; ^2^Department of Health Sciences, National Natural Science Foundation of China, 83 Shuangqing Road, Haidian Distract, Beijing 100085, China; ^3^Department of Oral BioSciences, Faculty of Dentistry, Prince Philip Dental Hospital, The University of Hong Kong, 34 Hospital Road, Hong Kong

## Abstract

This study was designed to evaluate the *in vitro* antifungal activities of four traditional Chinese medicine (TCM) extracts. The inhibitory effects of pseudolaric acid B, gentiopicrin, rhein, and alion were assessed using standard disk diffusion and broth microdilution assays. They were tested against six oral *Candida* species, *Candida albicans, Candida glabrata, Candida tropicalis, Candida krusei, Candida dubliniensis,* and *Candida guilliermondii*, including clinical isolates from HIV-negative, HIV-positive, and Sjögren's syndrome patients. It was found that pseudolaric acid B had the most potent antifungal effect and showed similar antifungal activity to all six *Candida* spp, and to isolates from HIV-negative, HIV-positive, and Sjögren's syndrome patients. The MIC values ranged from 16 to 128 *μ*g/mL. More interestingly, a synergistic effect of pseudolaric acid B in combination with fluconazole was observed. We suggest that pseudolaric acid B might be a potential therapeutic fungicidal agent in treating oral candidiasis.

## 1. Introduction

*Candida *species are commensal fungi that are found in 30–50% of human oral cavities. However, under certain conditions, commensal fungi can transform into opportunistic pathogens that cause both superficial mucosal and systemic mycoses. As the most common opportunistic infection, the incidence of oral candidiasis has increased rapidly for several decades due to the growing number of compromised hosts, such as patients with organ transplants or HIV infection or those undergoing chemotherapy or misusing antibiotics. In particular, oral candidiasis has much greater prevalence in immunocompromised individuals, such as patients with HIV infection (66%) [1] and Sjögren's syndrome (54.2–80%) [2, 3]. In addition, an important trend that can be detected with the incidence of oral candidiasis is an increase in the number of infections related to the non-albicans* Candida* species such as *Candida glabrata, Candida krusei, *and* Candida tropicalis*. This situation poses a clinical challenge because some non-albicans* Candida* species are inherently resistant to first-line antifungals such as fluconazole, especially in medically complex groups of patients [4, 5]. Therefore, novel fungal therapies for effective management of *Candida* infections are urgently required.

Research on traditional Chinese medicines (TCMs) or their extracts is known to be one important means of developing new drugs. Although some TCMs have already been demonstrated to possess activity against *Candida albicans* [6–8], their true anticandidal properties against most non-albicans* Candida *species remain unknown. In this study, we evaluated the anticandidal effect of four active constituents (pseudolaric acid B, gentiopicrin, rhein, and alion) extracted from TCMs that have been used to treat infectious diseases in China for thousands of years.

## 2. Materials and Methods

### 2.1. Organisms and Culture Conditions

Salivary specimens were collected from various groups of patients with oral candidiasis, including those with candidiasis alone and free from systemic disease, HIV infection, or Sjögren's syndrome. Specimens were obtained from Peking University Department of Oral Medicine. The study was approved by the Ethics Committee of Peking University Health Science Center.

*Candida* strains were isolated and stored in sterile water at 4°C and passaged two times on Sabouraud dextrose agar (SDA) before use. The identity of *Candida *species was confirmed using standard methodology: yeast-like growing colonies on SDA were routinely Gram-stained and, if found to be yeast cells by microscopy, were identified with the standard carbohydrate assimilation tests and with the API 20C AUX (bioMérieux, France) complemented with germ tube test and growth assessment at 45°C. The API 20C yeast identification system was inoculated with the samples, and the results were interpreted by following the manufacturer's instructions.

Six *Candida *species, *Candida albicans* (15 strains), *Candida glabrata* (4 strains), *Candida tropicalis* (4 strains), *Candida krusei* (4 strains), *Candida dubliniensis *(4 strains), and *Candida guilliermondii* (4 strains), were isolated and included in this study. *C. albicans* ATCC 90028 and *C. krusei* ATCC 6258 were used as quality control strains in all susceptibility testing experiments.

### 2.2. Preparation of TCM Compounds Suspensions

A total of four constituents of TCMs were obtained from the National Institute for the Control of Pharmaceutical and Biological Products of China: pseudolaric acid B (purity 99.1%), gentiopicrin, rhein, and alion (purity, ≥98%). The powder was dissolved in DMSO or distilled water at the appropriate concentrations. Fluconazole (kindly provided by Pfizer Pharmaceuticals, Pfizer-Roerig, Groton, CT, USA, purity, 99.7%) was dissolved in distilled water as a control. All drug solutions were stored in sterile tubes at −70°C and were further diluted in the assay medium before use.

### 2.3. Antifungal Susceptibility Testing

Two standard methods were employed to evaluate the fungicidal effect of TCM extracts as described in NCCLS M44-A [9] and M27-A2 [10].

### 2.4. Disk Diffusion Testing

All clinical strains were subcultured on SDA overnight, and inocula were adjusted to 0.5 McFarland standards (1 × 10^6^–5 × 10^6^ CFU/mL). Mueller-Hinton agar supplemented with 2% glucose and 0.5 *μ*g/mL methylene blue were used as described previously [11]. Plates were incubated automatically with a Spiral plater (Autoplate 4000; Spiral Biotech, Inc., Bethesda, MD, USA). Subsequently, filter paper discs (9 mm in diameter) containing 25 *μ*g of preprepared drugs were placed on the agar plates and incubated at 35°C for 24–48 hours. Meanwhile, discs containing 25 *μ*g fluconazole and solvent were included as positive and negative controls, respectively. Afterwards, the diameter of the inhibition zone was measured and zone inhibition interpretive criteria for fluconazole disc testing were based on NCCLS M44P recommended categories. The isolates with zone diameters ≥ 19 mm were reported as sensitive to fluconazole, whereas those with diameters of 15–18 mm and ≤14 mm were reported to have intermediate sensitivity and resistance to fluconazole, respectively [9]. Quality controls were performed with each batch of clinical isolates by testing *C. krusei* ATCC 6258 and *C. albicans* ATCC 90028 with a recommended acceptable performance range. The experiment was repeated on three separate occasions.

### 2.5. Broth Microdilution Susceptibility Testing

The TCM extract that showed antifungal activity in the agar diffusion assay was subsequently selected to determine the MIC. Broth microdilution susceptibility testing was performed according to the NCCLS M-27A criteria [10]. A spectrophotometer was used to prepare the inoculum to a concentration equivalent to 0.5 McFarland standard at 520 nm. The suspensions were further diluted in RPMI 1640 medium (Life Technologies, New York, NY, USA) to yield an inoculum concentration of approximately 10^4^ CFU/mL. The microdilution test was performed in presterilized, polystyrene, flat-bottom, 96-well microtiter plates, and each of the *Candida *species was exposed to a double dilution of each TCM extract. The microtiter plates were then incubated for 24 hours at 35°C, and the endpoints were read with a microtiter plate reader (SpectraMAX 340 Tunable Microplate Reader; Molecular Devices Ltd., Sunnyvale, CA, USA) at 520 nm. Testing of these isolates was performed in quadruplicate. The MIC of each TCM drug was defined as the lowest concentration at which there was 100% inhibition of yeast growth. According to CLSI criteria 100% growth inhibition is defined as clear wells. Therefore, the OD readings at MIC value were similar to the negative control without *Candida*. The MIC of fluconazole was determined for each species in parallel as a control, and antibiotic-free solvent was included as a negative control. MICs of fluconazole were determined at 50% inhibition (MIC50). To ensure reliable results, the MICs of the quality control strains *C. krusei* ATCC 6258 and *C. albicans* ATCC 90028 were monitored within the reference range.

### 2.6. Interaction of Pseudolaric Acid B and Fluconazole In Vitro

The interaction between pseudolaric acid B and fluconazole against five resistant *C. albicans *strains was tested using a microdilution checkerboard technique according to CLSI (formerly NCCLS) document M27-A2 (2002). Each drug was serially diluted twofold in RPMI 1640 medium. The final drug concentrations ranged from 128 to 0.125 *μ*g/mL for fluconazole and for pseudolaric acid B. Plates were incubated at 35°C for 24 hours. For pseudolaric acid B, the endpoint was determined as the lowest concentration to produce optically clear wells (no growth, MIC-0) or a cell count of ≤5% of the control well. For fluconazole, considering that the trailing phenomenon often occurs, the MIC was defined as the lowest concentration showing prominent 50% growth inhibition compared with the growth control. For the combination test, the MIC was defined as the lowest concentration showing prominent 100% growth inhibition. The fractional inhibitory concentration (FIC) for each drug was calculated by dividing the MIC in the presence of the second drug by the MIC in its absence, with the sum of the two FICs giving the FIC index (FICI). FICI values were interpreted as follows: FICI ≤ 0.5, synergy; 0.5 < FICI ≤ 4, no interaction; FICI > 4, antagonism.

### 2.7. Statistical Analysis

Statistical analysis was performed with SPSS for Windows version 10.0 (SPSS, Chicago, IL, USA). Continuous variables were analyzed by ANOVA test. All values were two tailed, and *P* < 0.05 was considered to indicate statistical significance.

## 3. Results

### 3.1. Disk Diffusion Assay

**Figure 1:**
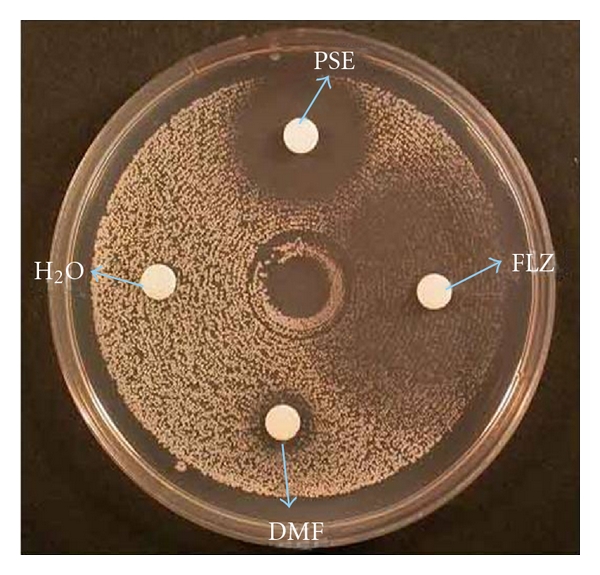
Agar diffusion assay of effect of pseudolaric acid B on *C. albicans* showing significant fungicidal activity. Note the circular zone is clearer compared to that of Fluconazole. PSE: pseudolaric acid B; FLZ: fluconazole; DMF: dimethylformamide; H_2_O: distilled water.

From the initial screening with disk diffusion assay, only pseudolaric acid B exhibited considerable anticandidal activity. It had zones of growth inhibition ranging from 8 to 25 mm against *C. albicans, C. glabrata, C. krusei, C. tropicalis, C. dubliniensis, *and* C. parapsilosis* (Figure 1). Gentiopicrin, rhein, and alion did not exert anticandidal activity and had no inhibitory zone. Thus, pseudolaric acid B had potent antifungal activity against all six evaluated *Candida *species.

### 3.2. Microdilution Susceptibility Test

The M-27A broth microdilution assay indicated that pseudolaric acid B had a strong inhibitory effect on all six *Candida *species, and the MIC ranged from 16 to 128 *μ*g/mL, with a mean of 40.58 ± 22.46 *μ*g/mL (geometric mean titer ± standard deviation) (Table 1). Pseudolaric acid B demonstrated an approximately similar level of antifungal activity against all the *Candida *species tested compared with that of fluconazole. ANOVA tests indicated no significant difference in MICs against different *Candida *species (*P* = 0.58 > 0.05). Also of interest were the clear, nontrailing endpoints for pseudolaric acid B against all *Candida* strains. This might be evidence that pseudolaric acid B is fungicidal, rather than fungistatic against *Candida *species. We evaluated the antifungal effect of pseudolaric acid B against *C. albicans* isolated from various sources including patients with Sjögren's syndrome (5 strains), HIV-negative patients (5 strains), and HIV-positive patients with oral candidiasis (5 strains) (Table 2). Pseudolaric acid B was equally effective against all the isolates tested (*P* = 0.34 > 0.05). More importantly, it demonstrated a strong anticandidal effect against fluconazole-resistant strains.

### 3.3. Interaction of Pseudolaric Acid B and Fluconazole

**Figure 2:**
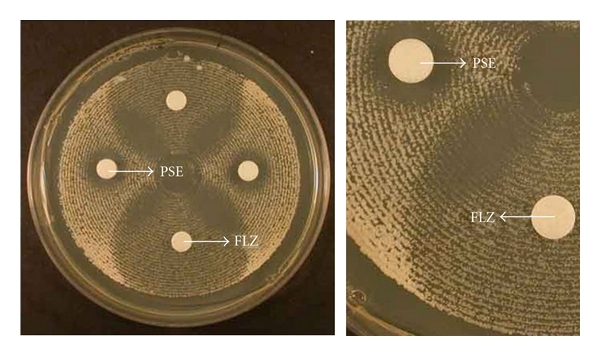
Agar diffusion assay of effect of pseudolaric acid B and fluconazole on *Candida albicans* demonstrated synergistic antifungal activity. Note that there is no microcolony growth in the cross-sectional area of pseudolaric acid B and fluconazole. PSE: pseudolaric acid B; FLZ: fluconazole.

A synergistic phenomenon was observed in the agar diffusion assay (Figure 2), which was confirmed by the checkerboard method. It was most pronounced after 24 hours incubation and was sustained through 48 hours. The results for the tested drug alone and in combination against the 14 isolates are summarized in Table 3. Pseudolaric acid B had *in vitro* antifungal activity against orally isolated *Candida *spp. More importantly, FICI showed a synergism of pseudolaric acid B and fluconazole against azole-resistant clinical isolates of *C. albicans*, whereas there was no such reaction with other *Candida* species.

## 4. Discussion

In recent years, candidiasis has reemerged with higher prevalence and mortality rates that are nearly 45% among compromised population groups [12]. Moreover, clinicians have encountered the new challenge of failure of treatment with existing antifungal agents. This may be due to either emergence of non-albicans species such as *C. krusei *and* C. glabrata*, which are more resistant to commonly used antifungals. Therefore, there is an urgent need for new antifungal agents for the efficient management of candidal infections. Research on the active constituents of natural or traditional medicines, which are a potential source for new drugs, is drawing more attention. The aim of our study was to examine the anticandidal effect of four constituents of TCMs, which have been used for antifungal treatment for thousands of years in China.

In the initial screening with the disc diffusion assay, pseudolaric acid B showed considerable zones of growth inhibition. The remaining three TCM extracts, gentiopicrin, rhein, and alion, did not exert a significant anticandidal effect, although the plants (*Gentian*, *Radix et Rhizoma Rhei,* and *Aloe*) from which they were isolated have been traditionally used to treat fungal skin infections. Pseudolaric acid is extracted from “tujingpi” or Cortex pseudolaricis, a TCM. To date, these natural products and their derivatives have been reported to exhibit antifungal, antifertility, cytotoxic, and antiangiogenic activities [13]. Although tujingpi has been used in China for the treatment of fungal skin infection since the 17th century, recent, studies on TCM extracts have led to the identification of the pseudolaric acids as the main antifungal constituents, in which pseudolaric acid B is one of the major antifungal components. Subsequent *in vitro* studies have shown that pseudolaric acid B is active against *C. albicans*, *Torulopsis petrophilum*, *Trichophyton mentagrophytes,* and *Microsporum gypseum *[14, 15]. However, the anticandidal effect of pseudolaric acid B against non-*Candida *species has rarely been reported, and there have been no reports of its activity against orally isolated *Candida *species. The purpose of the present study was therefore to investigate activity of pseudolaric acid B against oral *Candida*, with an emphasis on non-albicans and* Candida *spp. from different sources.

It is well known that fungal infection is much more common in immunocompromised individuals, such as HIV-positive and Sjögren's syndrome patients. Furthermore, there are increasing reports on *C. albicans* that is resistant to antifungal medications especially in HIV-infected hosts and Sjögren's syndrome patients who have undergone repeated courses of antifungal therapy [4, 16]. Therefore, *C. albicans *strains isolated from HIV-infected and Sjögren's syndrome patients were included as part of our study. We found that pseudolaric acid B was effective against *C. albicans* isolated from HIV-positive, HIV-negative, and Sjögren's syndrome patients, including both fluconazole-sensitive and -resistant strains. This potent nonselective effect of pseudolaric acid B implies that it has potential as a novel antifungal agent, especially against clinically resistant infections.

More interestingly, pseudolaric acid B demonstrated approximately similar antifungal activity against *C. albicans* and non-albicans* Candida*. This phenomenon is of great importance, because susceptibility to antifungal medication differs significantly among *Candida *spp. For example, *C. krusei* has natural resistance to fluconazole, a standard antifungal agent commonly used in the clinic. Another example is *C. glabrata,* which possesses a low level of intrinsic resistance to the azole drugs and fluconazole and ketoconazole. The antifungal activity of pseudolaric acid B against different *Candida *spp. demonstrated in the present study may provide a possible answer to the frustrating drug resistance and warrants further research.

In this study, the phenomenon of the interactions between pseudolaric acid B and fluconazole was observed by agar diffusion assay and determined qualitatively by checkerboard microdilution method. The FICI model is the most commonly used approach to study the interaction between antifungal drugs and has been used to interpret the data. It was found that a combination of pseudolaric acid B and fluconazole exhibited good synergism against oral azole-resistant isolates of *C. albicans*, which has not been reported previously. It is well established that the development of antifungal drug resistance in *C. albicans* follows from prolonged exposure to azole antifungals [17, 18]. In patients with advanced AIDS, oral candidiasis continues to be a common presenting illness associated with significant morbidity. In recent years, oral fluconazole, given its low toxicity, has become the most common form of treatment for symptomatic oral candidiasis [19]. The widespread use of fluconazole has, however, led to an increased incidence of clinically resistant oral candidiasis due to infection with fluconazole-resistant organisms. The finding of synergy in the present study was significant because azole resistance is an emerging issue and it is necessary to find an effective and novel therapeutic method to overcome the problem of drug resistance.

## 5. Conclusions

In conclusion, the present study provided strong evidence of the potent antifungal activity of pseudolaric acid B against a wide range of *Candida *species, and the synergistic effect of pseudolaric acid B in combination with fluconazole. Further studies are warranted to explore the mechanism behind the antifungal activity of pseudolaric acid B.

## Figures and Tables

**Table 1:** MIC of pseudolaric acid B against *Candida* species.

*Species*	Total Number	PSE MIC/MFC	FLZ MIC
*C. tropicalis*	4	32.00 ± 20.13*	1.19 ± 3.63
*C. krusei*	4	53.82 ± 16.00	53.82.00 ± 16.00
*C. dubliniensis*	4	53.82 ± 16.00	1.68 ± 3.47
*C. glabrata*	4	32.00 ± 20.13	11.37 ± 12.38
*C. guilliermondii*	4	32.00 ± 20.13	3.36 ± 1.00
*C. albicans*	15	42.22 ± 26.68	11.06 ± 37.76
*C. k.* ATCC 6258	1	64	32 ~ 64
*C. a* ATCC 90028	1	32	0.5 ~ 1

MIC: minimum inhibitory concentration; MFC: minimum fungicidal concentration; PSE: pseudolaric acid B; FLZ: fluconazole. *Data were demonstrated as geometric mean titer ± SD.

**Table 2:** MIC of pseudolaric acid B against *C. albicans* strains isolated from different sources.

Species	Isolate no.	PSE (*μ*g/mL) MIC/MFC	PSE (mmol/L)	FLZ (*μ*g/mL)	
				MIC50	MFC
*C. albicans*	SS	32	0.074	64	128
*C. albicans*	SS	32	0.074	128	256
*C. albicans*	SS	32	0.074	64	128
*C. albicans*	SS	32	0.074	64	128
*C. albicans*	SS	32	0.074	32	128
*C. albicans*	HIV(−)	64	0.148	2	64
*C. albicans*	HIV(−)	32	0.074	0.5	32
*C. albicans*	HIV(−)	64	0.148	2	64
*C. albicans*	HIV(−)	64	0.148	8	128
*C. albicans*	HIV(−)	32	0.074	4	64
*C. albicans*	HIV(+)	128	0.296	16	128
*C. albicans*	HIV(+)	32	0.074	64	128
*C. albicans*	HIV(+)	32	0.074	4	128
*C. albicans*	HIV(+)	32	0.074	8	128
*C. albicans*	HIV(+)	64	0.148	2	64

MIC: minimum inhibitory concentration; MFC: minimum fungicidal concentration; PSE: pseudolaric acid B; FLZ: fluconazole; SS: Sjögren's syndrome.

**Table 3:** *In vitro* interaction of pseudolaric acid B and fluconazole (1 : 1) against oral Candida spp.

Strain	Agent	MIC(*μ*g/mL)		FIC	FICI	Outcome
		Alone	Combination			
*C. albicans (M3)*	FLZ	64	1	1/64	<0.5	Synergy
	PSE	32 (0.074)*	1	1/32		
*C. krusei (K2)*	FLZ	16	16	1	0.5–4	No interaction
	PSE	64 (0.148)	16	16/64		
*C. tropicalis (T1)*	FLZ	0.25	0.25	1	0.5–4	No interaction
	PSE	16 (0.037)	0.25	0.25/16		
*C. dubliniensis (D4)*	FLZ	32	32	1	0.5–4	No interaction
	PSE	64 (0.148)	32	32/64		
*C. glabrata (gp)*	FLZ	0.5	0.5	1	0.5–4	No interaction
	PSE	32 (0.074)	0.5	0.5/32		
*C. guilliermondii (gm3)*	FLZ	2	2	1	0.5–4	No interaction
	PSE	32 (0.074)	2	2/32		

MIC: Minimum inhibitory concentration; FIC: fractional inhibitory concentration; FICI: fractional inhibitory concentration index; PSE: pseudolaric acid B; FLZ: fluconazole; *M, K2, T1, D4, gp, gm3: *strain no. *Concentration in micromolar.
